# An algorithm for constructing the skeleton graph of degenerate systems of linear inequalities

**DOI:** 10.1371/journal.pone.0175819

**Published:** 2017-04-13

**Authors:** José Manuel Méndez Martínez, Jesús Urías

**Affiliations:** Instituto de Física, Universidad Autónoma de San Luis Potosí, San Luis Potosí, SLP, México; Nankai University, CHINA

## Abstract

Derive the quantitative predictions of constraint-based models require of conversion algorithms to enumerate and construct the skeleton graph conformed by the extreme points of the feasible region, where all constraints in the model are fulfilled. The conversion is problematic when the system of linear constraints is degenerate. This paper describes a conversion algorithm that combines the best of two methods: the incremental slicing of cones that defeats degeneracy and pivoting for a swift traversal of the set of extreme points. An extensive computational practice uncovers two complementary classes of conversion problems. The two classes are distinguished by a practical measure of complexity that involves the input and output sizes. Detailed characterizations of the complexity classes and the corresponding performances of the algorithm are presented. For the benefit of implementors, a simple example illustrates the stages of the exposition.

## Introduction

Mathematical modeling in areas of science such as the physics of quantum nonlocality [1–7] and systems biology [8–11], frequently take the form of a system of linear inequalities in some Euclidean space. Every inequality in the system defines a half-space and the intersection of all half-spaces constitutes the polyhedral feasible region of the model. In physics, the no-signaling approach to quantum nonlocality [2–4, 7] leads to degenerate systems of linear constraints for the correlations between the parties playing in a correlational set up [4, 6, 7]. In systems biology, the structural analysis approach for the mathematical modeling of a biological complex applies constraint-based methods that take the form of degenerate linear systems of inequalities [8–11].

In order to transform the model constraints into quantitative predictions, conversion methods are necessary to derive the relational structure (a skeleton graph or a network) that is conformed by the set of extreme points of the feasible region. In the no-signaling approach to quantum nonlocality, the extreme points of the polytope of correlations provide the local operations and the elementary non-local Popescu-Rohrlich channels [2–4] which are necessary (and in any cases sufficient) to simulate any no-signaling correlational scenario [5]. Quite similarly, the “elementary constituents”, “archetypes” or “modes” in biology [12], are provided by the extreme points of the feasibility region. In both cases, the resulting output descriptions display a wide range of structural complexities, characterized by measurements as graph entropy [13, 14] and graph similarity [15].

The most effective conversion methods available arise from combinatorial geometry [16–19]. However, when the half-space descriptions of the extreme points of the feasible polytope are degenerate a combinatorial explosion is produced that is the cause of stubborn difficulties for their enumeration [19–23].

The paper introduces an algorithm to convert a half-space description into the skeleton graph conformed by the set of extreme points of the feasible region. Using a combination of incremental [18, 19] and pivotal [24, 25] methods, an algorithm with a good performance to resolve degeneracy and to complete the traversal of the set of extreme points is presented. The effects of degeneracy are studied computationally for a very large number of half-space descriptions, organized into four families according to the degree of degeneracy of the input half-space descriptions and on the complexity of the output graphs.

The standard formulation of a constraint-based model is the system of linear inequalities

Ax≤b. (1)

Matrix *A* is real and of size *m* × *n*. The model constraints determine the entries of *A* and the entries of vector $b\in R^{m}$. Each one of the *m* constraint inequalities in Eq (1) defines a half-space of the euclidean space of dimension *n*, *E*_*n*_.

Every row of matrix *A* is the constraint vector $a_{i}\in R^{n}$, with index i∈I={0,…,m−1}. Vector a_*i*_ defines the *i*-th feasible half-space $H_{i}=\left\{x\in R^{n}:a_{i}\cdot x\leq b_{i}\right\}$. The intersection of all half-spaces i∈I constitutes the set of feasible values of $x\in R^{n}$, which conform the convex polytope $P=\{x\in R^{n}:Ax⩽b\}$. We assume *P* is bounded and of full affine dimension *n*, for which is necessary that *m* > *n*. The description of *P* that is provided by Eq (1) is known as a half-space description, or *H*-description.

However, what is physically meaningful is the combinatorial structure that is encoded in the skeleton graph *G*(*P*) of *P*, known as the *V*-description of *P*. The conversion of the *H*-description Eq (1) into the graph *G*(*P*) is accomplished when the set of vertices *V* = {p ∈ *P*: p is extreme} and the set of edges *E* ⊂ *V* × *V* have been determined. Then, the skeleton graph *G*(*P*) = (*V*, *E*) discloses the organizational structure that is implicit in the set of linear constraints Eq (1).

Whether p is a vertex of *G*(*P*) is decided by the non-negativity of its slack vector *s*(p) = *b* − *A*p and the rank of its set of active hyperplanes $Z\left(p\right)=\left\{i\in I:a_{i}\cdot p=b_{i}\right\}$. First, a point p is in the feasible region *P* whenever the slack vector *s*(p) is non-negative. Then, p ∈ *P* is an extreme point if, and only if, rank $(Z(p))=\text{dim}(\text{span}\{a_{i}:i\in Z\left(p\right)\})=n$. An extreme point p is regular (or non-degenerate) if it has an active set Z(p) of cardianlity |Z(p)|=n. The active set of a regular extreme point p, Z(p), is a basis. Otherwise, when |Z(p)|=n+σ and *σ* ≥ 1, the extreme point p is *σ*-degenerate.

The combinatorial triviality of regular extreme points does not present any difficulty to the simplex pivoting rules [16, 24, 25]. However, when pivoting around a *σ*-degenerate extreme point p, the method faces up to (n+σn) potential bases in the active set Z(p), so the exhaustive search of the neighboring points of p requires the examination of (n+σn) simplex tableaus and a factible pivote has to be looked for by testing the *n* entries of *m* − *n* − *σ* rows of every tableau. The misery then is that a *σ*-degenerate vertex demands the simplex method to do a search among a multiplicity of

μ=n(m-n-σ)n+σn (2)

alternatives, just for finding the neighboring points of p. The amount of searches Eq (2) may be—per vertex!—much larger than the total number of vertices of the complete skeleton graph *G*(*P*). Besides, when doing the search, the simplex method may get trapped in an endless cycle or just get stalled [22]. Several approaches have been tried out to overcome such deficiencies [20–23].

The practice of the double description method has proved its efficiency [18, 19] in the determination of the extreme rays of highly degenerate polyhedral cones [26]. However, the method is not efficient for the construction of the complete skeleton graph of large degenerate systems of linear inequalities, mainly due to the very large number of tentative vertices that are produced at intermediate stages of the conversion procedure [26]. The majority of intermediate vertices are discarded at the end. To overcome this situation, we have designed a swift and compact pivoting method to determine the neighbors of extreme points, by taking as the input the extreme rays of a cone. In this way we have combined in Algorithm 5 the best of two methods: the incremental slicing of cones to defeat degeneracy [18, 19], and pivoting around extreme points for a swift traversal of extreme points [24].

The incremental procedure goes slicing a cone, starting with a regular cone that is broader than and includes the target cone. The preparation of the base cone to be the input of the incremental procedure is explained in Section 1: a basis is chosen from the active set Z(p) and a standard algebraic method produces the extreme rays of the corresponding regular cone. The half-spaces in the active set Z(p) that are not part of the basis set are inserted by the incremental procedure, one-by-one, until they are exhausted and the target cone has been sculpted. The half-space insertion procedure is explained in Section 2. The explanation includes (*i*) the alternative combinatorial or algebraic test necessary to identify the 2-face cuts during the slicing procedure and (*ii*) a recording strategy that helps the algorithm to reduce the number of tests.

The extreme rays that are produced by the incremental procedure provide the scanning directions for the pivoting rule that is followed to determine the set of neighboring points of p. The pivoting rule is developed in Section 3. In Section 4 the incremental and pivoting methods are combined in Algorithm 5, which converts the system of linear inequalities into the skeleton graph conformed by the set of extreme points of the feasible region.

The computational practice in Section 5 affords understanding about the effects that the degeneracy present in the input systems has on the performance of Algorithm 5. The very large number of input systems employed in the practice of Section 5 is organized in four families that offer a controlled and distributed sampling of the complexity spectrum of the conversion problem. The amount adopted to estimate the complexity combines the average degeneracy 〈*σ*〉 that is present in the input system and the average connectivity *κ* of the output graph *G*(*P*).

The family with the lowest complexity consists of regular (non degenerate) polytopes with *H*-descriptions produced at random [7]. The other three are one-parameter families. The family with the highest complexity consists of Birkhoff polytopes [27, 28]. The other two families, with intermediate complexities, consist of no-signaling polytopes [7]. Section 5 details the characterization of the four families, produced by applying Algorithm 5.

The computational practice of Section 5 distinguishes two classes of conversion problems. A first class consists of systems of linear inequalities that have a combined complexity which becomes smaller as a function of the input size. The systems in this class convert into skeleton graphs with a number of vertices that grows faster than their vertex-connectivity, as a function of the input size. For these conversion problems (I) the CPU time consumed by Algorithm 5 is mostly applied to complete the traversal of extreme points and not to resolve degeneracy, (II) the algebraic test for 2-face cuts in Algorithm 5 is faster than the combinatorial test and (III) the incremental procedure is highly sensitive (*i*) to the choice of the input basis set and (*ii*) to the insertion order of the cutting half-spaces.

The second class of conversion problems distinguished by the computational practice in Section 5 has a combined complexity that does not decrease as a function of the input size. As the input size of the systems of linear inequalities in this class is increased, the number of vertices of their skeleton graphs increases and the vertex-connectivity does not decrease. For these conversion problems (I) the CPU time consumed by Algorithm 5 is mostly applied to resolve the degeneracy present at the *H*-description and not to complete the traversal of extreme points, (II) the combinatorial test for 2-face cuts in Algorithm 5 is faster than the algebraic test and (III) the incremental procedure is not sensitive (*i*) to the choice of the input basis set and (*ii*) neither to the insertion order of the cutting half-spaces.

The two complementary classes described above are detailed in Sections 5 and 6.

For the benefit of the implementor we make use of a simple, but rich enough, no-signaling constraint *H*-description [7] as example to illustrate our exposition.

### Example (Outset)

The conversion problem consists of the two-party correlations that are feasible for a no-signaling box [4] with a binary input per party and asymmetric in its outputs, producing one out of 3 and of 2 possible outcomes per party respectively. The no-signaling and non-negativity constraints on correlations [7] produce the system of linear inequalities in Table 1. The feasible polytope *P*_NS_ is the intersection of *m* = 24 half-spaces in an Euclidean space of dimension *n* = 14.

**Table 1:** Parameters (*A*, *b*) of the heuristic example.

$a_{i}=-{\hat{x}}_{i}\;,\;i\in \left\{0,\ldots ,13\right\}$	$a_{14}=-{\hat{x}}_{0}-{\hat{x}}_{1}-{\hat{x}}_{2}+{\hat{x}}_{5}+{\hat{x}}_{6}$
$a_{15}=-{\hat{x}}_{9}-{\hat{x}}_{10}-{\hat{x}}_{11}+{\hat{x}}_{12}+{\hat{x}}_{13}$	$a_{16}=-{\hat{x}}_{0}-{\hat{x}}_{3}+{\hat{x}}_{9}$
$a_{17}=-{\hat{x}}_{1}-{\hat{x}}_{4}+{\hat{x}}_{10}$	$a_{18}=-{\hat{x}}_{5}-{\hat{x}}_{7}+{\hat{x}}_{12}$
$a_{19}=-{\hat{x}}_{6}-{\hat{x}}_{8}+{\hat{x}}_{13}$	$a_{20}={\hat{x}}_{0}+{\hat{x}}_{1}+{\hat{x}}_{2}+{\hat{x}}_{3}+{\hat{x}}_{4}$
$a_{21}={\hat{x}}_{0}+{\hat{x}}_{1}+{\hat{x}}_{2}+{\hat{x}}_{7}+{\hat{x}}_{8}$	$a_{22}={\hat{x}}_{0}+{\hat{x}}_{1}+{\hat{x}}_{3}+{\hat{x}}_{4}+{\hat{x}}_{11}$
$a_{23}={\hat{x}}_{5}+{\hat{x}}_{6}+{\hat{x}}_{7}+{\hat{x}}_{8}+{\hat{x}}_{9}+{\hat{x}}_{10}+{\hat{x}}_{11}-{\hat{x}}_{12}-{\hat{x}}_{13}$	
$b_{i}=\left\{\begin{array}{cc} 0, & i\in \{0,\ldots ,19\}\\ 1, & i\in \{20,\ldots ,23\} \end{array}\right.$	

Each vector ${\hat{x}}_{i}$ (*i* = 0 to 13) is the standard *i*-th unit vector of Euclidean space *E*_14_.

Using the constraint vectors in Table 1 one verifies that the origin p = 0 is an extreme point of *P*_NS_ since its active set Z(p)={0,…,19} has rank (Z(p))=14 and the slack vector at p = 0, *s*(0) = b − A p = b, is not negative. This extreme point is degenerate with σ=|Z(p)|-n=6 and for the simplex method it represents a multiplicity Eq (2) of *μ* = 2,170,560 search options. This huge value of *μ* is to be compared with the 6 cutting half-spaces that the double description method needs to insert, one at a time.

## 1 Regular cones as the base case

The cone described by the active set Z(p) of the extreme point p is the set $K_{Z(p)}=\left\{x\in R^{n}:a_{i}\cdot x\leq b_{i},\;i\in Z\left(p\right)\right\}$. The cone $D_{Z(p)}:=\left\{x\in R^{n}:a_{i}\cdot x\leq 0,i\in Z\left(p\right)\right\}$ is the translation of cone $K_{Z(p)}$ to the origin,

$$K_{Z(p)}=p+D_{Z(p)}\;.$$ (3)

Since rank (Z(p))=n, both cones $K_{Z(p)}$ and $D_{Z(p)}$ are peaked, with apices located at p and the origin, respectively. The polyhedral cone $K_{Z(p)}$ fits the feasible region *P* and the 1-faces (or extreme rays) of $K_{Z(p)}$ provide the directions to scan for the neighbors of p. Then, and in view of Eq (3), the first step towards the skeleton graph *G*(*P*) is to convert the half-space description of cone $D_{Z(p)}$ into its set of extreme rays $X_{Z(p)}$, such that $D_{Z(p)}=\{r=\sum_{\rho \in X_{Z(p)}}c_{\rho }\;\rho :\;c_{\rho }\geq 0\}$.

The determination of the set of extreme rays of a degenerate cone is the subject matter of the next section. Meanwhile, the extreme rays of a regular cone may be obtained from its half-space description by methods of linear algebra. A regular cone $D_{B}$ is the intersection of the half-spaces of a basis B⊊Z(p). The set $X_{B}$ of extreme rays of $D_{B}$ is given in the following.

**Lemma 1.1** (Regular cones). *Let* p *be an extreme point of* *P*. *Let* B⊊Z(p) *be a basis. The set of extreme rays of the cone* $D_{B}$ *is*

$$X_{B}=\left\{\rho_{j}\in R^{n}:a_{i}\cdot \rho_{j}=-\delta_{ij},\;i,\;j\in B\right\}\;.$$ (4)

The set $X_{B}$ in Lemma 1.1 is the negative of the biorthogonal companion of B. Given that B⊊Z(p) we have that $D_{B}⊃D_{Z(p)}$. The set of extreme rays of cone $K_{B}$ is the set $X_{B}+p$.

For a degenerate vertex p with active set Z(p) the double description method, discussed in the next section, produces the set $X_{Z(p)}$ of extreme rays of cone $D_{Z(p)}\subset D_{B}$ by starting with the set of rays provided by Lemma 1.1 for a basis B⊊Z(p).

### 1.1 Example (The base cone)

The extreme point p = 0 of the no-signaling polytope *P*_NS_ has degeneracy *σ* = 6. The basis B={0,…,13}⊊Z(p), through lemma 1.1, provides us with the set of rays

$$X_{B}=\left\{\rho_{i}={\hat{x}}_{i}:i=0,\ldots ,13\right\}\;.$$ (5)

Use the constraint vectors in Table 1 to verify the membership conditions of set Eq (4).

## 2 Incremental slicing of cones

The incremental procedure to generate the set of extreme rays $X_{Z(p)}$ of the cone at a degenerate extreme point p, which is described by the set of active planes Z(p), starts with the approximate set $X_{B}$, provided by lemma 1.1 for a basis B⊊Z(p). The set $B^{\prime }≔Z\left(p\right)\backslash B$ is not empty by degeneracy. Then, the half-spaces remaining in $B^{\prime }$ are introduced one at a time. The insertion process produces a non-increasing chain of cones by eliminating the current extreme rays which are not in the feasible half-space introduced and adding the new extreme rays that are created by the half-space that has been added. When all hyperplanes in the degeneracy set $B^{\prime }$ have been inserted, the set of extreme rays $X_{Z(p)}$, describing cone $D_{Z(p)}$, is produced.

Assume the insertion procedure has gone adding half-spaces from $B^{\prime }$ as far as to generate $\text{cone}\left(X_{J^{\prime }}\right)\subset D_{B}$, for some $J^{\prime }⊊Z\left(p\right)$. Assume the procedure is to advance one step farther by adding the half-space *H*_*k*_, for some *k* remaining in $Z\left(p\right)\backslash J^{\prime }$. Then, by working on the current set of rays $X_{J^{\prime }}$ and the vector a_*k*_ that is associated to *H*_*k*_, the procedure will produce the set of extreme rays $X_{J}$ that results from the insertion of the half-space *H*_*k*_. This one-plane insertion procedure defines the function $(X_{J^{\prime }},a_{k})\mapsto X_{J}=F(X_{J^{\prime }},a_{k})$.

The procedure represented by **F** begins with the partition of the current set of rays $X_{J^{\prime }}$ into three sets. A first set collects the rays that are within the feasible half-space *H*_*k*_, the set $X^{+}≔\left\{\rho \in X_{J^{\prime }}:a_{k}\cdot \rho <0\right\}$. A second set is $X^{-}≔\left\{\rho \in X_{J^{\prime }}:a_{k}\cdot \rho >0\right\}$, which is the set of rays outside the feasible half-space *H*_*k*_. The third set is $X^{0}≔\left\{\rho \in X_{J^{\prime }}:a_{k}\cdot \rho =0\right\}$, which collects the rays lying on the hyperplane ∂*H*_*k*_. The rays in *X*^+^∪*X*^0^ remain extreme for the next cone $D_{J}$. We have $X_{J}⊇X^{+}\cup X^{0}$. Rays in *X*^−^ become unfeasible, but they are necessary to complete the set of extreme rays of cone $D_{J}$.

New extreme rays in $X_{J}$ are the intersections of the hyperplane ∂*H*_*k*_ with 2-faces of the current cone $D_{J^{\prime }}$. In order to decide whether a pair of extreme rays *ρ* and *ρ*′ constitute a 2-face of $D_{J^{\prime }}$ or not, let $J^{\prime }|\rho =\left\{i\in J^{\prime }:a_{i}\cdot \rho =0\right\}$ and let $J^{\prime }|(\rho ,\rho^{\prime })=J^{\prime }\left|\rho \cap J^{\prime }\right|\rho^{\prime }$ be the joint active subset of the pair (*ρ*, *ρ*′) in $D_{J^{\prime }}$. Central to the incremental slicing procedure [7, 19] is the following.

**Lemma 2.1** (Tests of colaminarity [19]). *For some* J⊊Z(p) *with* rank(J)=n, *let* *ρ* *and* *ρ*′ *be extreme rays of* $D_{J}$. *The following statements about the pair* (*ρ*, *ρ*′) *are equivalent*.

1. *The pair* (*ρ*, *ρ*′) *constitues a 2-face of* $D_{J}$.
2. $\text{rank}(J|(\rho ,\rho^{\prime }))=n-2$.
3. *Let* *φ* *be an extreme ray of* *D*_*J*_. *If* J|φ⊃J|(ρ,ρ′), *then either* *φ* = *ρ* *or* *φ* = *ρ*′.

**Algorithm 1** The standard insertion method.

**def F** $\left(X_{J^{\prime }}\;,\;a_{k}\right)$:

1 $X^{+}≔\left\{\rho \in X_{J^{\prime }}:\rho \cdot a_{k}<0\right\}$

2 $X^{-}≔\left\{\rho \in X_{J^{\prime }}:\rho \cdot a_{k}>0\right\}$

3 $X^{0}≔\left\{\rho \in X_{J^{\prime }}:\rho \cdot a_{k}=0\right\}$

4  *X* = *X*^+^ ∪ *X*^0^

5  **for** *ρ*′ ∈ *X*^−^:

6   **for** *ρ* ∈ *X*^+^:

7    **if** *ρ* ∼ *ρ*′:      // Lemma 2.1

8     *X* = *X* ∪ {*φ*_*k*_(*ρ*′, *ρ*)} // Formula (6)

9  **return** *X*

When statement (*a*) holds we say that the pair of extreme rays (*ρ*, *ρ*′) is colaminar in $D_{J}$ and denote the relation by *ρ* ∼ *ρ*′.

The completion of $X_{J}$ is achieved by incorporating all the intersections that the current hyperplane ∂*H*_*k*_ makes with the 2-faces of $D_{J^{\prime }}$ that are framed by pairs of rays (*ρ*′, *ρ*)∈*X*^−^ × *X*^+^. When the case is that *ρ* ∼ *ρ*′, the ray

$$\varphi_{k}\left(\rho^{\prime },\rho \right)=\left(a_{k}\cdot \rho^{\prime }\right)\rho -\left(a_{k}\cdot \rho \right)\rho^{\prime }$$ (6)

is extreme for the sliced cone $D_{J}=D_{J^{\prime }}\cap H_{k}$. The collection of all such rays,

$$Y_{k}=\left\{\varphi_{k}\left(\rho^{\prime },\rho \right):\left(\rho^{\prime },\rho \right)\in X^{-}\times X^{+}\;\text{and}\;\rho ∼\rho^{\prime }\right\}\;,$$ (7)

completes the set of extreme rays of $D_{J}$, $X_{J}=X^{+}\cup X^{0}\cup Y_{k}$. The pair $\left(J,X_{J}\right)$ constitutes a double description of the cone $D_{J}$.

The test of colaminarity in Eq (7), corresponding to line 7 of the pseudo-code for function **F** in Algorithm 1, may proceed in one of two standard ways. Either by applying the algebraic test (*b*) in lemma 2.1, which applies methods of linear algebra, or the combinatorial test (*c*), which runs over the set $X_{J^{\prime }}\backslash \left\{\rho ,\rho^{\prime }\right\}$ [19, 29].

### 2.1 Example (A first plane insertion)

The extreme point p = 0 of *P*_NS_ has active set Z(p)={0,…,19}. The set $X_{B}$ of extreme rays of the base cone $D_{B}$, for basis B={0,…,13}, was determined in Eq (5). There remains $B^{\prime }=\{14,\ldots ,19\}$ half-spaces to be inserted. By inserting *H*_14_ first, the current set $X_{B}$ is partitioned into the following subsets,

$$\begin{array}{cc} X^{+} & =\{\rho_{0}={\hat{x}}_{0}\;,\;\rho_{1}={\hat{x}}_{1}\;,\;\rho_{2}={\hat{x}}_{2}\}\;,\\ X^{-} & =\{\rho_{5}={\hat{x}}_{5}\;,\;\rho_{6}={\hat{x}}_{6}\}\;,\\ X^{0} & =\{\rho_{3}={\hat{x}}_{3}\;,\;\rho_{4}={\hat{x}}_{4}\;,\;\rho_{7}={\hat{x}}_{7}\;,\;\ldots \;,\;\rho_{13}={\hat{x}}_{13}\}\;. \end{array}$$ (8)

For the base cone $D_{B}$ the test of colaminarity for the pairs in *X*^−^ × *X*^+^ may be skipped, because in a regular cone, as is the case for $D_{B}$, every pair of extreme rays is colaminar (Lemma 2.2 below). The set of new rays *Y*_14_ that is obtained by applying Formula (6) to every pair in *X*^−^ × *X*^+^ is shown in Table 2. The new cone in the chain $D_{J^{\prime }}\subset D_{B}$, with $J^{\prime }=\{0,\ldots ,13,14\}$, has gained six new rays and lost the two rays in *X*^−^, which became unfeasible.

**Table 2:** Set of newly produced rays *φ*_*i*_ in Example 2.1.

$\rho_{0}∼\rho_{5}\to \varphi_{0}={\hat{x}}_{0}+{\hat{x}}_{5}$, $\rho_{1}∼\rho_{5}\to \varphi_{1}={\hat{x}}_{1}+{\hat{x}}_{5}$, $\rho_{2}∼\rho_{5}\to \varphi_{2}={\hat{x}}_{2}+{\hat{x}}_{5}$
$\rho_{0}∼\rho_{6}\to \varphi_{3}={\hat{x}}_{0}+{\hat{x}}_{6}$, $\rho_{1}∼\rho_{6}\to \varphi_{4}={\hat{x}}_{1}+{\hat{x}}_{6}$, $\rho_{2}∼\rho_{6}\to \varphi_{5}={\hat{x}}_{2}+{\hat{x}}_{6}$

Rays produced by the intersection of *H*_14_ with every pair in *X*^−^ × *X*^+^ that is colaminar, *ρ*′ ∼ *ρ*.

The set $X_{Z(p)}$ of extreme rays of the degenerate cone $D_{Z(p)}$ is produced by the iteration of the insertion function **F** in Algorithm 1. The half-spaces in the degeneracy set $B^{\prime }=Z\left(p\right)\backslash B$ are introduced one by one until the set is exhausted. This incremental slicing of cones constitutes function **X** in Algorithm 2. The base case to start the iteration, line 1 of Algorithm 2, is the set of extreme rays $X_{B}$ of a basis B⊊Z(p), as is given by lemma 1.1.

**Algorithm 2** Incremental slicing with standard insertion function **F**.

**def X** (B, $B^{\prime }$):

1  $X=X_{B}$   // Lemma 1.1

2  **for** $k\in B^{\prime }$:

3   *X* = **F**(*X*, a_*k*_)  // Algorithm 1

4  **return** *X*

### 2.2 Example (Standard incremental slicing)

For the extreme point p = 0 of *P*_NS_ the standard slicing function *X* is applied to basis B={0,…,19}, with complement $B^{\prime }=Z\left(p\right)\backslash B=\{14,\ldots ,19\}$. The first half-space *H*_14_ was inserted in section 2.1 already.

When the next three half-spaces in {15, 16, 17} are inserted, all pairs in *X*^−^ × *X*^+^ pass the test of colaminarity, producing each a new extreme ray for the subsequent cone in the chain. Consequently, the number of intermediate extreme rays goes up. It is not so for the insertion of the last two half-spaces, *H*_18_ and *H*_19_. The 2-faces of the current cone that are slashed by the insertion of *H*_18_ are identified by the test of colaminarity. This time the subset of colaminar pairs in *X*^−^ × *X*^+^, shown in Table 3, is rather sparse. The last two insertions, *H*_18_ and *H*_19_, make the number of intermediate rays come down. Upon conclusion, the standard function *X* in Algorithm 2 returns a set of 54 extreme rays for the cone $D_{Z(p=0)}$, which is almost four times the 14 extreme rays at a non-degenerate extreme point in dimension 14.

**Table 3:** Colaminar pairs of rays (2-faces) being slashed by the insertion of half-space *H*_18_ in Example 2.2.

	$\rho_{7}^{\left(B\right)}$	$\rho_{0}^{\left(14\right)}$	$\rho_{1}^{\left(14\right)}$	$\rho_{2}^{\left(14\right)}$	$\rho_{2}^{\left(16\right)}$	$\rho_{10}^{\left(16\right)}$	$\rho_{2}^{\left(17\right)}$	$\rho_{10}^{\left(17\right)}$
$\rho_{2}^{\left(15\right)}$	∼	∼	∼	∼				
$\rho_{4}^{\left(16\right)}$	∼							
$\rho_{5}^{\left(16\right)}$	∼		∼	∼				
$\rho_{7}^{\left(16\right)}$	∼							
$\rho_{4}^{\left(17\right)}$	∼							
$\rho_{5}^{\left(17\right)}$	∼	∼		∼				
$\rho_{7}^{\left(17\right)}$	∼							

The set *X*^+^ runs along the top line and the set *X*^−^ goes down along the left column.

The computational practice has shown [19, 26] that the typical behaviour of the number of intermediary rays during the slicing process is to go up and then come down. At the step corresponding to Table 3 of Example 2.2, the elements of *X*^−^ listed along the left column of Table 3 become unfeasible but they give rise to as many new rays as colaminarity symbols ∼ are entered in Table 3. Although a great deal of the intermediary rays computed by function **X** in Algorithm 2 do not survive as extreme rays of the target cone $D_{Z(p)}$, the standar slicing function **F** has to apply the test of colaminarity (either (*b*) or (*c*) in lemma 2.1) to *every pair* in the current set *X*^−^ × *X*^+^ and at *every step* in the chain leading to $X_{Z(p)}$. The standard incremental slicing procedure, Algorithms 2 and 1, gets bogged down in applying an excess of colaminarity tests.

Unfortunately, we do not have a method to avoid the tests of colaminarity during the slicing procedure. What we have is a method to reduce the number of times the test is applied. The method is simple but improves considerably the CPU time of the slicing procedure. The idea is to combine a record of pairs of rays we know are colaminar, as to avoid a re-testing of pairs, with a necessary condition of colaminarity. If the necessary condition is not fulfilled, the pair is rejected and that is it. Otherwise, the pair is searched in the record. If it is not found, then the colaminarity test is applied to the pair. After a positive test, a new ray is produced and the pair is included in the record of colaminar pairs. This improvement is implemented in Algorithm 3.

**Algorithm 3** Incremental cone slicing with 2-face recording and rejection test.

**def X′** (B, $B^{\prime }$):

1  $X=X_{B}$                // Lemma 1.1

2  *L* = *X* × *X*                 // Lemma 2.2

3  **for** $H\in B^{\prime }$:

4   produce the sets *X*^+^, *X*^0^, *X*^−^

5   *X* = *X*^0^ ∪ *X*^+^

6   **for** (*ρ*′, *ρ*)∈*X*^−^ × *X*^+^:

7    **if** $|J|(\rho^{\prime },\rho )|<n-2$:       // Lemma 2.3

8     **continue**

9    **if** (*ρ*′, *ρ*)∈*L*:

10     *X* = *X* ∪ {*φ*_*H*_(*ρ*′, *ρ*)}          // Formula (6)

11     replace (*ρ*, *ρ*′) in *L* by (*ρ*, *φ*_*H*_)

12     continue

13    **else if** *ρ*′ ∼ *ρ*:             // Lemma 2.1

14     *X* = *X* ∪ {*φ*_*H*_(*ρ*′, *ρ*)}          // Formula (6)

15     *L* = *L* ∪ {(*ρ*, *φ*_*H*_)}

16  **return** *X*

The record of pairs of colaminar rays in Algorithm 3 is initialized in line 2 to $X_{B}\times X_{B}$ because the following.

**Lemma 2.2** (For a basis all pairs are colaminar). *Let* B⊊Z(p) *be a basis. The set of 2-faces of cone* $D_{B}$ *is* $X_{B}\times X_{B}$.

The improved Algorithm 3 rejects a pair as colaminar when the following condition does not hold.

**Lemma 2.3** (A minimum is required). *Let* (*ρ*, *ρ*′) *be a pair of extreme rays of cone* $D_{J}$, *for some* J⊂Z(p) *of* rank(J)=n. *If* *ρ* ∼ *ρ*′, *then* $|J|(\rho ,\rho^{\prime })|>n-3$.

The minimum condition in Lemma 2.3 is tested in lines 7 and 8 of Algorithm 3.

The incremental method we have described for the enumeration of extreme rays of cones is not effective when extended for the conversion of the complete system of linear inequalities. A weakness of the method is that the number of intermediary vertices can grow exponentially as compared to the number of true vertices. That is why we follow a pivoting method instead.

## 3 Getting the neighbouring extreme points

The rays in the set $X_{Z(p)}$ (computed by Algorithm 3) define the directions to scan around p, looking for its neighboring extreme points. Assuming the feasible region *P* is compact, the positive linear span of every $\rho \in X_{Z(p)}$ necessarily intersects one of the hyperplanes in I\Z(p): the set of hyperplanes $N_{p}\left(\rho \right)=\left\{t\in I\backslash Z\left(p\right):a_{t}\cdot \rho >0\right\}$ is not empty. Then, for $t\in N_{p}\left(\rho \right)$ the hyperplane ∂*H*_*t*_ is pierced by *ρ* at the point q_*t*_ = p + *ρ*λ_*t*_, with

$$\lambda_{t}=\left(b_{t}-p\cdot a_{t}\right)/\left(\rho \cdot a_{t}\right)\;>0.$$ (9)

The point q_*t*_ with the smallest (positive) *λ*_*t*_ is the neighboring extreme point of p along *ρ*.

**Lemma 3.1** (Pivoting around p [7]). *For every* $\rho \in X_{Z(p)}$ *let* $N_{p}\left(\rho \right)$ *be as defined above. The set of neighboring extreme points of* p *is*

$$V_{p}=\left\{q=p+\lambda \rho :\;\rho \in X_{Z(p)},\;\lambda =\min_{t\in N_{p}\left(\rho \right)}\left\{\lambda_{t}\right\}\right\}\;.$$

The unordered pair {p, q}, for every q ∈ *V*_p_, is an element of the set of edges of p, denoted by *E*_p_. Lemma 3.1 is the core of the pivoting function **P** that returns the set *V*_p_ of neighbors and the set *E*_p_ of edges of p. The function **P** is defined by the pseudo-code listed in Algorithm 4.

**Algorithm 4** Pivoting to get the neighbours.

**def P** (B, $B^{\prime }$):

1  *V*_p_ = ∅; *E*_p_ = ∅

2  *X* = **X**′(B, $B^{\prime }$)            // Algorithm 3 either 2

3  **for** *ρ* ∈ *X*:

4   $N≔\{t\in I\backslash Z\left(p\right):\;\rho \cdot a_{t}>0\}$

5   $\lambda =\min_{t\in N}\{\left(b_{t}-p\cdot a_{t}\right)/\left(\rho \cdot a_{t}\right)\}$  // Lemma 3.1

6   q = p + *λ* *ρ*

7   *V*_p_ = *V*_p_ ∪ {q}; *E*_p_ = *E*_p_ ∪ {(p, q)}

8 **return** *V*_p_, *E*_p_

### 3.1 Example (A neighbouring extreme point)

The extreme point p = 0 of *P*_NS_ has active set Z(p)={0,…,19}. The hyperplanes not going thorough p are in the complement I\Z(p)={20,21,22,23}. For the ray $\rho ={\hat{x}}_{0}\in X_{Z(p=0)}$ we have that $a_{23}\cdot {\hat{x}}_{0}=0$ while for $i\in N_{p=0}\left({\hat{x}}_{0}\right)=\{20,21,22\}$ we have that a_*i*_ · *ρ* = 1. Using Eq (9) we obtain *λ*_20_ = *λ*_21_ = *λ*_22_ = 1, so that the three hyperplanes in $N_{p=0}\left({\hat{x}}_{0}\right)$ intersect with the positive span of ray ${\hat{x}}_{0}$ at the point $q=p+\lambda {\hat{x}}_{0}={\hat{x}}_{0}$.

## 4 Assembling the skeleton graph

The traversal of extreme points starts from a known extreme point p of *P*, which becomes the first vertex of the skeleton graph *G*(*P*). The set of vertices *V* is initialized as {p}. The connectivity of p in *G*(*P*) is determined by function **P** in Algorithm 4 by providing us with two sets: the set of neighboring vertices *V*_p_ and the set of edges *E*_p_. The set of edges *E* of the skeleton is initialized as *E*_p_. All the neighboring vertices of p are awaiting to be scanned. They take a place in the queue *Q*, initialized with the set *V*_p_. With this provision, an exhaustive search proceeds by applying repeatedly the following three steps, finishing when there are no vertices awaiting in the queue.

1. Pick the next vertex v that is available in *Q* and remove v from *Q*.
2. Apply function **P** to vertex v in order to generate the sets *V*_v_ and *E*_v_.
3. Update the set of edges *E* = *E* ∪ *E*_v_, the set of scanned vertices *V* = *V* ∪ {p}, and the queue of vertices awaiting to be scanned, *Q* = *Q* ∪ (*V*_v_ \ *V*).

When the procedure stops, the pair (*V*, *E*) is the graph *G*(*P*). The procedure defines function **G**, which is shown in Algorithm 5.

**Algorithm 5** Assembling the skeleton graph *G*(*P*).

**def G** (B, $B^{\prime }$):

1  *V*_p_, $E_{p}=P\left(B,B^{\prime }\right)$                   // Algorithm 4

2  *Q* = *V*_p_; *E* = *E*_p_; *V* = {p}

3  **while** *Q* ≠ ∅:

4   Pop out next v ∈ *Q* and update *Q* = Q \ {v}

5   Choose a basis $B_{v}$ and let $B_{v}^{\prime }=Z\left(v\right)\;\backslash \;B_{v}$

6   *V*_v_, $E_{v}=P\left(B_{v},B_{v}^{\prime }\right)$                 // Algorithm 4

7   *E* = *E* ∪ *E*_v_; *V* = *V* ∪ {v}; *Q* = *Q* ∪ {*V*_v_ \ *V*}

8  **return** *V*, *E*

### 4.1 Example

Function **G** in Algorithm 5 starts at the extreme point p = 0 to produce the skeleton graph *G*(*P*_*NS*_). The output graph has 108 vertices and 1,548 edges, which represent a 27% of the edges of the complete graph *K*_108_. Two thirds (72) of the 108 vertices are sparsely connected, having degree 16. The other 36 vertices are densely connected, having degree 54 (connecting with a half of all the vertices in the graph). To appreciate how densely connected is the output graph *G*(*P*_NS_), compare the 108 vertices it has against the upper bound 16,016 for regular polytopes described by the same number of half-spaces and in the same Euclidean space [30].

## 5 Computational practice

Algorithm 5 converts systems of linear inequalities into the skeleton graph *G*(*P*) of the feasible polytope *P*. The combinatorial complications introduced by degeneracy are explored computationally on a wealth of systems of linear inequalities, organized in four families of varying complexity.

In our scheme the input systems to Algorithm 5 are sized by the product variable *ζ* = *nm*〈*Z*〉, which includes (*a*) the dimension (or number of constrained variables) *n*, (*b*) the number *m* of half-spaces (or constraints) and (*c*) the average number 〈*Z*〉 = *n* + 〈*σ*〉 of active hyperplanes of the extreme points of the feasible region *P*. The size of the output graph *G*(*P*) is measured by (*a*) the number of vertices |*V*| of the graph and (*b*) its connectivity *κ* = 〈*X*〉/|*V*|, where 〈*X*〉 is the average number of edges attached to a vertex of *G*(*P*). The number of vertices |*V*| weights the bulk of the graph and the connectivity *κ* < 1 is a simple measure of the complexity of the graph’s topology. The two output variables, *κ* and |*V*|, and the input size *ζ* provide a practical characterization of the complexity of a conversion problem, including degeneracy and its consequences.

**Fig 1:**
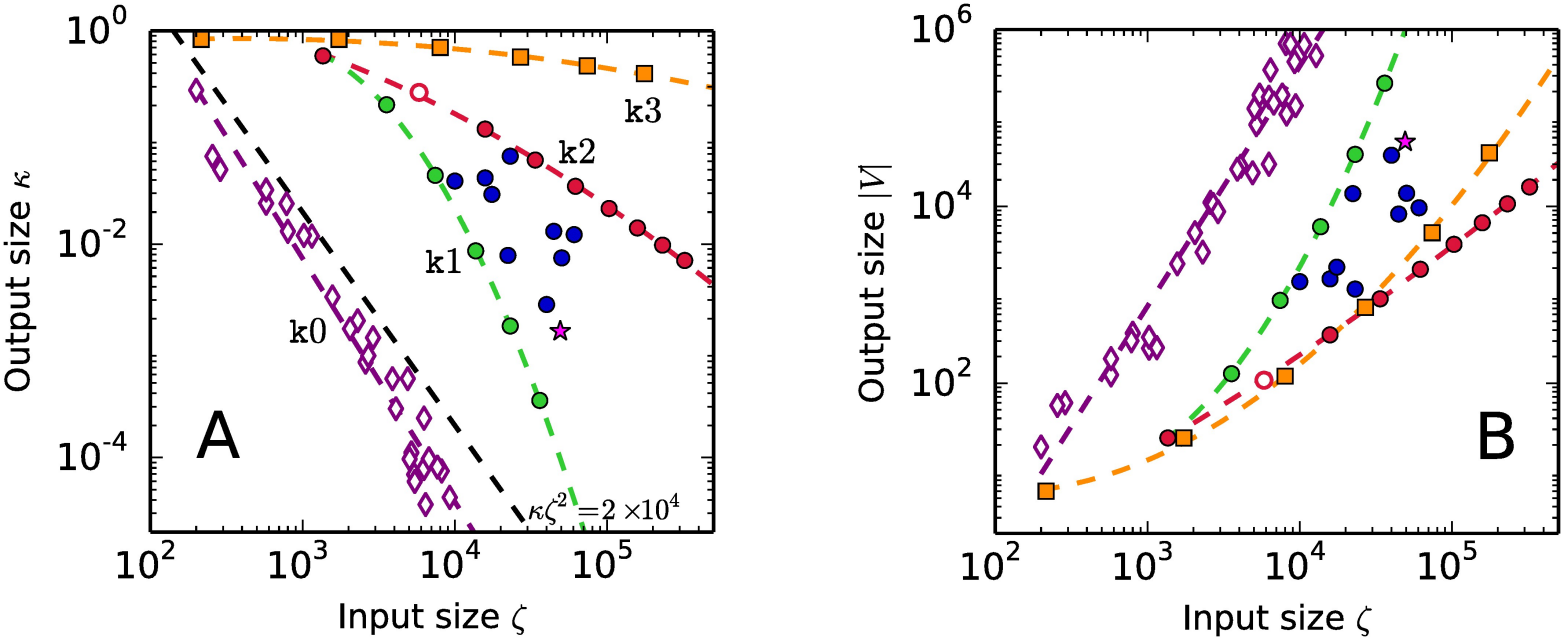
Output-input features of the systems of linear inequalities used in the computational practice. The variable *κ* is the average connectivity of the output graph, |*V*| is the number of vertices and *ζ* is the input size, which includes the amount of degeneracy.

Algorithm 5 translates the input size into some form of complexity of the output graph. Extreme simplicity is reached by the family of non-degenerate half-space descriptions that converts into regular graphs. The regular systems we are including in our practice have *m* constraint hyperplanes tangent to the (*n* − 1)-sphere in the Euclidean space $E_{n}$. The points of tangency were generated at random and equally distributed on the positive ortant of the sphere. The family of regular systems labeled k0 in Fig 1 (represented by hollow diamonds) covers the ranges 7 ≤ *n* ≤ 20 and 16 ≤ *m* ≤ 64. The random systems so produced are regular with probability 1. For any value of the input size *ζ*, the non-degenerate systems of linear inequalities produce the bulkiest, Fig 1B, and most sparsely connected, Fig 1A, output graphs. The trend followed in Fig 1A by the non-degenrate k0 family implies that degenerate half-space descriptions have combined output-input size values *κζ*^2^ ≥ 2 × 10^4^.

The zones of intermediate complexity in Fig 1A and 1B are populated by bipartite no-signaling system [7], represented by circular dots. The star is the tripartite no-signaling binary system [6]. The bipartite system with the smallest input size *ζ* corresponds to Bell’s experimental setup [1] and the hollow dot is the heuristic example in Table 1. The upper sequence of no-signaling systems in Fig 1A (red online) |referred to as the k2 family| emerges from Bell’s setup by increasing the number *v* of outcomes for one of the parties only. The output graph has |*V*| = 2*v*^2^(2 + (*v* − 1)^2^) [7].

The lower sequence of no-signaling systems in Fig 1A (green online) |referred to as the k1 family| emerges from Bell’s setup by increasing the number of input options available to one of the parties. The degenerate systems in the k1 family produce the bulkiest but most sparsely connected class of no-signaling graphs.

Each sequence of no-signaling systems in Fig 1 (red and green online) constitutes a one-parameter family. The blue dots dispersed between the two border sequences correspond to binary systems differing from Bell’s setup in at least two parameters. Fig 1 confirms that the no-signaling polytopes constitute a good example of highly degenerate half-space descriptions that produce output graphs with a large volume |*V*| and a moderately dense connectivity *κ*.

Birkhoff systems of half-spaces produce densely connected skeleton graphs, which appear in the upper part of the output-input map of Fig 1A. They are referred to as the k3 family of *ℓ* × *ℓ* doubly-stochastic matrices [*p*_*ij*_] and are represented by square dots in Fig 1 (orange online). The polyhedral region *P*_*ℓ*_ of doubly-stochastic matrices is known as Birkhoff’s polytope. It is a notable polytope in various branches of mathematics [27, 28]. A polytope *P*_*ℓ*_ is the feasible region, in Euclidean space of dimension *n* = (*ℓ* − 1)^2^, of the non-negativity constraints *p*_*ij*_ ≥ 0, subjected to the normalization conditions $\sum_{i=1}^{\ell }p_{ij}=\sum_{i=1}^{\ell }p_{ji}=1$. The graph *G*(*P*_*ℓ*_) has |*V*| = *ℓ*! vertices [27]. The output-input maps in Fig 1 show that Birkhoff’s polytopes have a highly degenerate half-space description and for high values of the input size *ζ* their output graphs become bulky while keeping a dense connectedness (the diameter of *G*(*P*_*ℓ*_) is 2 for every *ℓ* [27]). The one-parameter k3 family of Birkhoff’s polytopes constitute our most complex exemplar.

**Fig 2:**
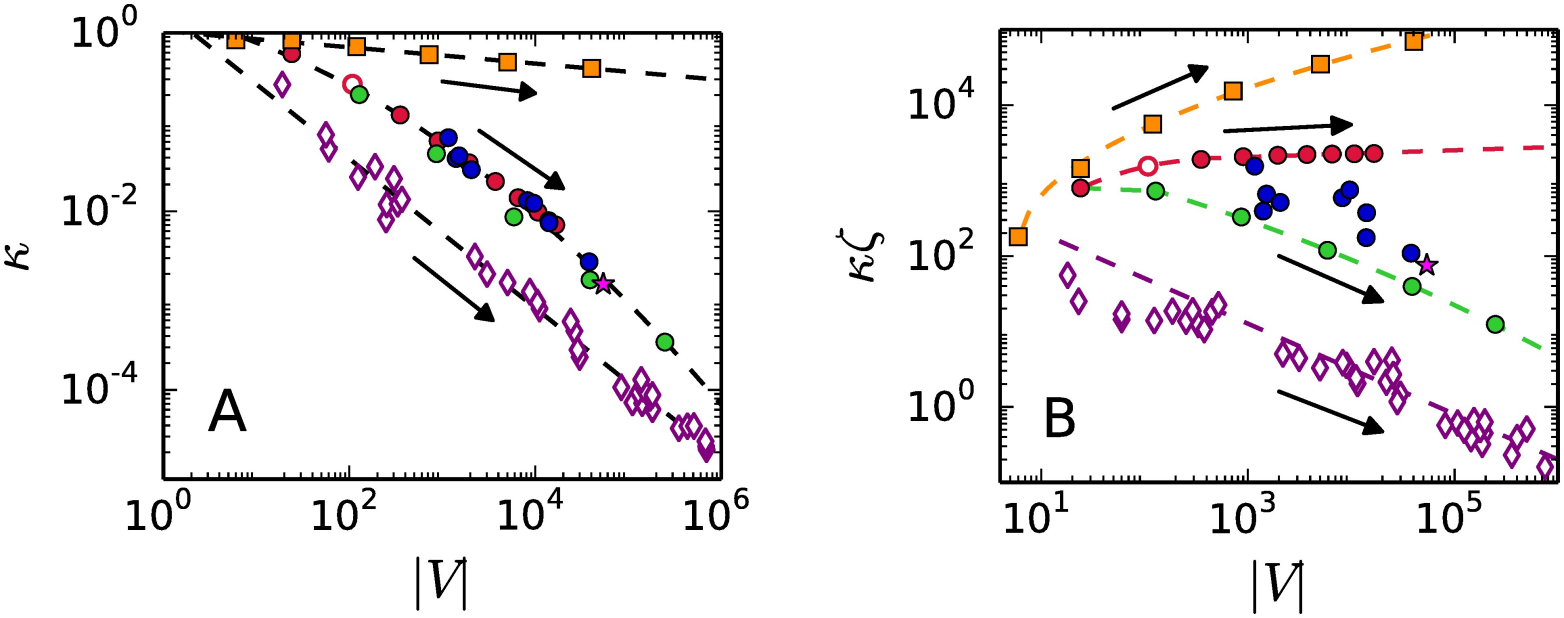
Complexity of the output graphs. A.- By volume size |*V*| and connectivity *κ*. B.- By volume size |*V*| and combined output-input size *κζ*. The arrows—in all figures—point to larger values of the input size *ζ*.

Fig 2A shows the practical estimate of complexity of the output graph *G*(*P*) that is afforded by volume |*V*| and connectivity *κ*. Each family follows a well defined “complexification path”. The arrows in the figures indicate the direction the input size *ζ* of the half-space descriptions becomes greater. The general trend, as the input size gets bigger, is an increase of the volume |*V*| at the cost of loosing the connectivity *κ*: degeneracy at the input is converted into connectivity at different rates per family. This fact is better appreciated in Fig 2B by using the combined output-input size *κζ*, instead of simply *κ*. The output graphs of the k1 family of systems exhibit a considerable increase in volume but degeneracy is not producing a dense connectivity. Fig 2B shows that family k2 exhibits the slowest growing rate of the volume of the graph and the combined output-input size *κζ* is maintained around the value *κζ* ∼ 2.1 × 10^3^. This constant value of *κζ* presents itself as a boundary between families.

**Fig 3:**
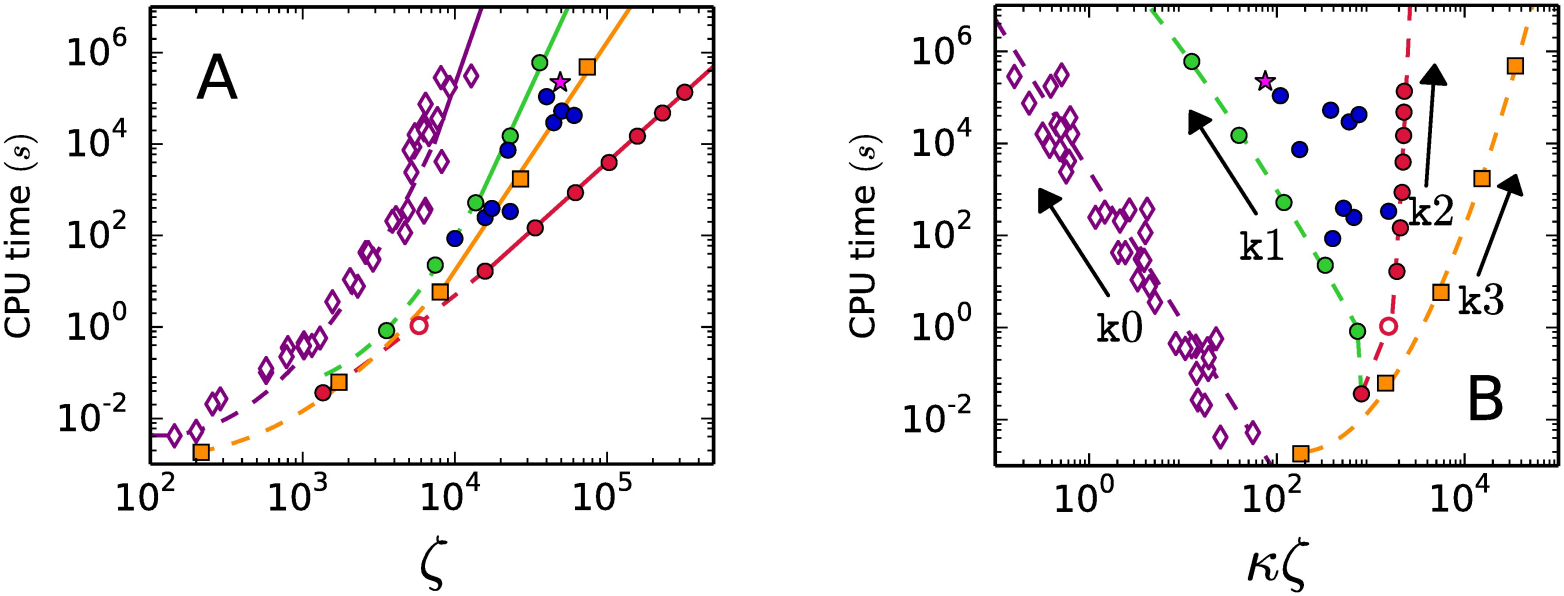
CPU time taken by Algorithm 5 to output the skeleton graph. A.- As a function of the input size *ζ*. B.- As a function of the combined output-input product *κζ*.

The time Algorithm 5 takes to output the graph of a regular system is orders of magnitude longer than the time needed to produce the graph of a degenerate system of the same input size *ζ*. For large values of *ζ* the CPU time for regular systems is $O(\zeta^{9.6})$, shown as a solid segment in Fig 3A.

In Fig 3A Algorithm 5 shows the best performance for the k2-family of no-signaling systems. The k2 family reaches in our exploration the biggest values of the input size *ζ* ∼ 10^6^ and the CPU time fits the law $O(\zeta^{3})$ exactly. At *ζ* ∼ 10^4^, Algorithm 5 consumes a CPU time to produce the skeleton graph of a k2-family degenerate system that is five orders of magnitud shorter than the CPU time needed for a regular system. The CPU times for the k1 and k3 families go, for large values of *ζ* in Fig 3A, as $O(\zeta^{7.2})$ and $O(\zeta^{5})$, respectively.

Differences in performance of Algorithm 5 are better appreciated in terms of the combined output-input variable *κζ*. Fig 3B shows a clear distinction between the four families of systems. Families k0 and k1 produce the most sparsely connected graphs and exhibit a similar growing rate of the CPU times as the combined variable *κζ* gets smaller. Algorithm 5 shows the opposite behavior on systems of family k3: the CPU time grows with *κζ*. In conoclusion, Algorithm 5 has a better performance on degenerate families with densely connected graphs |families k2 and k3.

The conversion algorithm involves two procedures that manage complex inputs. One is in line 6 of Algorithm 5 that resolves degeneracy to determine (*a*) the extreme rays of cones and (*b*) the neighboring extreme points. The other procedure is the exhaustive traversal of extreme points. The traversal procedure is Algorithm 5 itself, excluding the time employed by line 6. Next test decides whether the CPU time consumed by Algorithm 5 is either employed in the resolution of degeneracy or in traversing the set of extreme points.

**Fig 4:**
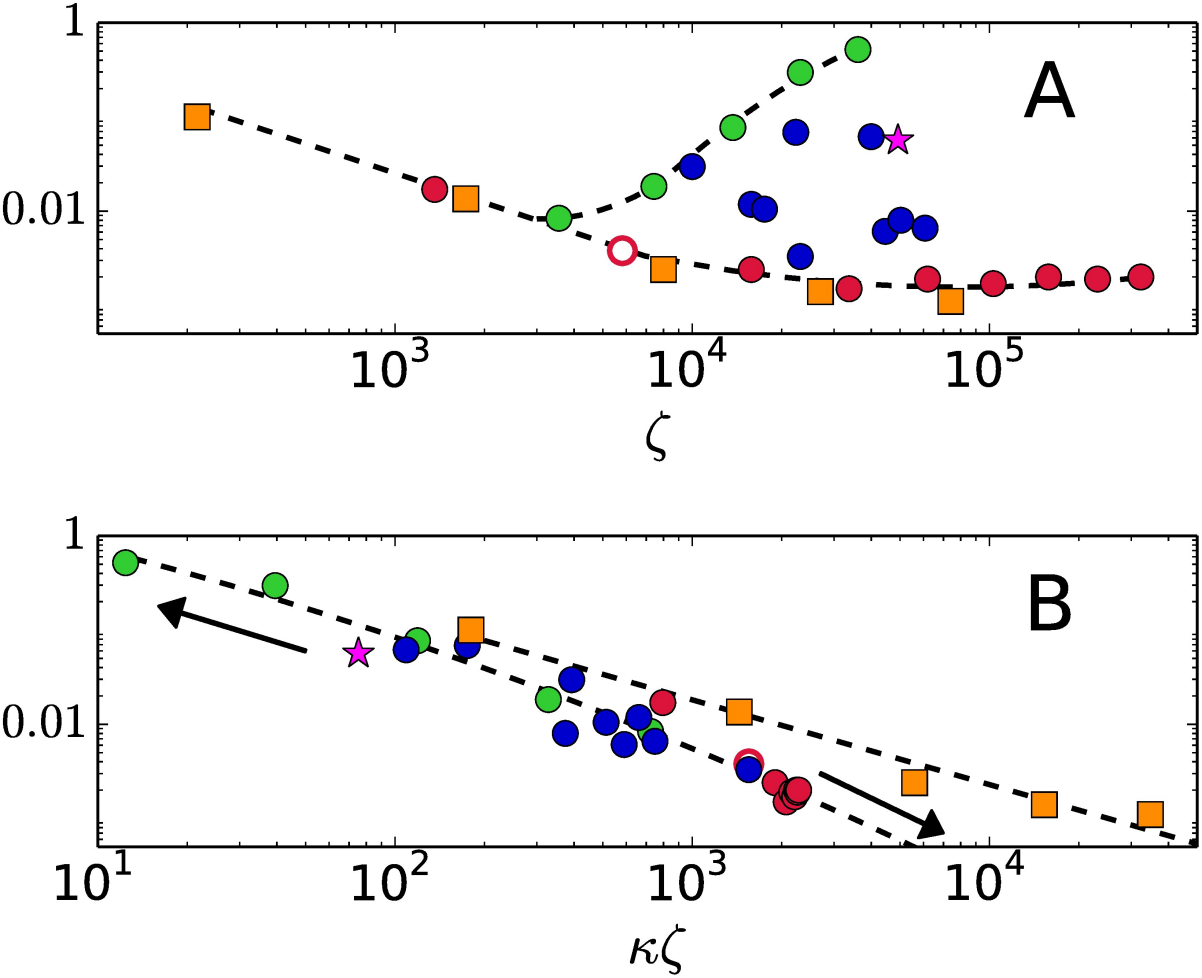
Fraction of CPU time consumed by Algorithm 5 to traverse the set of extreme points. A.- As a function of the input size *ζ*. B.- As a function of the combined output-input size *κζ*.

The fraction of total CPU time employed to traverse the set of extreme points is shown in Fig 4A as a function of the input size *ζ*. Degenerate systems with a densely connected graph are the dots following the lower slashed line in Fig 4A. For systems with large values of *ζ* but with a sparse connectivity *κ*, the traversal of extreme points takes longer than to resolve degeneracy. The systems following the upper slashed line in Fig 4A have a huge amount of sparsely connected vertices.

Considering the combined variable *κζ*, the fraction of time employed in the traversal is shown to decreases monotonously in Fig 4B, the arrows point to larger values *ζ*. The traversal time is not greater than a 10% of the CPU time for systems with values of *κ* *ζ* ≳ 100. For systems of the k2-family Algorithm 5 takes only a 0.17% of the time to complete the traversal: 99.83% of the time is taken by degeneracy. The conclusion is that complexity rests entirely upon the degeneracy of the conversion problem when *κζ* > 100, regardless of the number of vertices |*V*|.

Degeneracy is the source of a combinatorial explosion of the search universe of simplex-based methods [25]. In contrast, degeneracy furnishes the incremental procedure with a huge set of options for the input $\left(B,\;B^{\prime }\right)$ in lines 5 and 6 of Algorithm 5. The alternatives consist in choosing a basis B⊂Z(p), an order for the *n* elements in B and an order for the *σ* elements in $B^{\prime }=Z\left(p\right)\backslash B$.

In our computational exploration we found that the incremental procedure is not sensitive to the order given to the elements of any chosen basis B. While for highly degenerate systems with less than moderately connected output graphs the choice of a basis B and the order adopted for $B^{\prime }$ are critical. The example system in Table 1 (in dimension *n* = 14) will be used to illustrates the situation.

**Fig 5:**
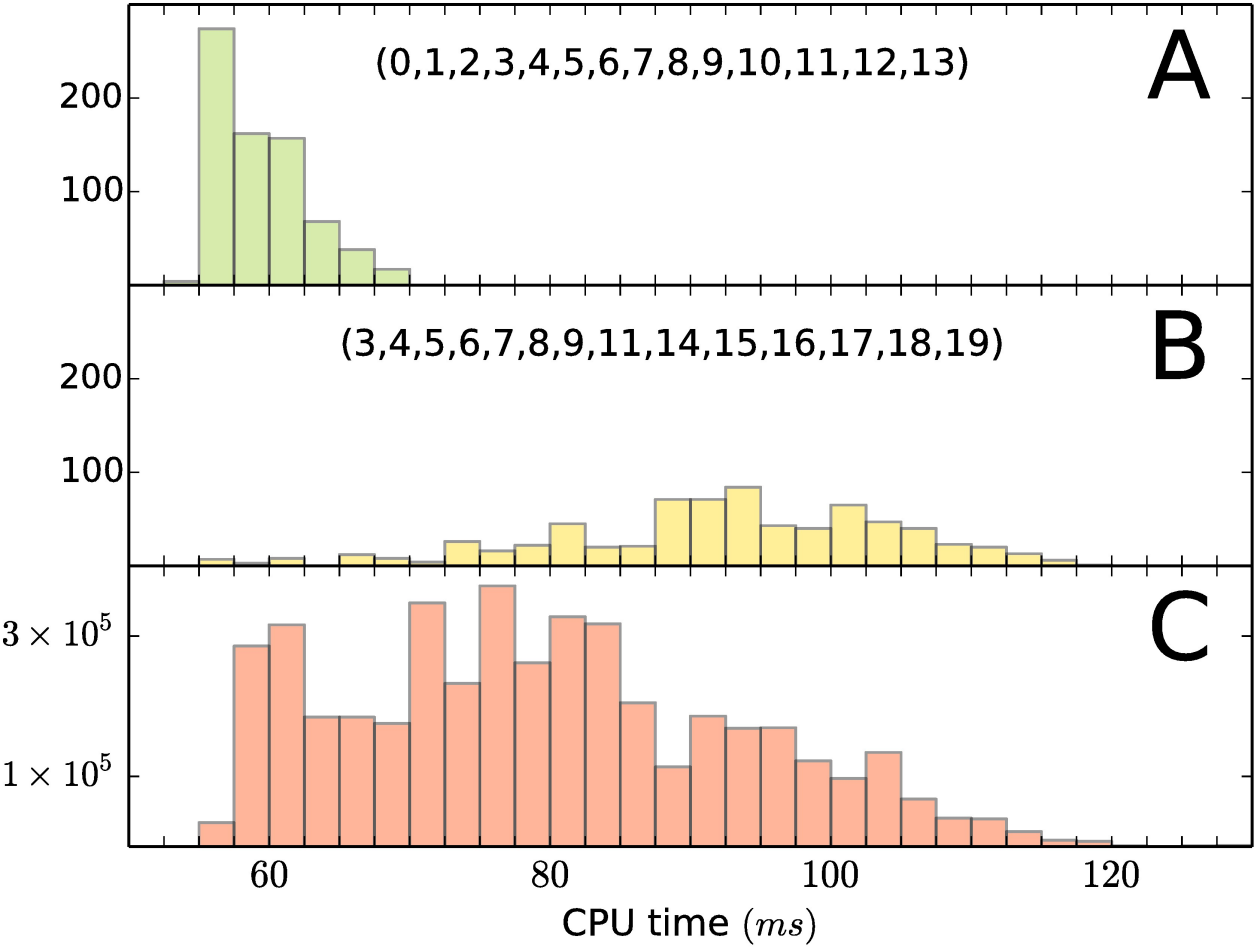
Distribution of insertion orders over CPU time. A.- Distribution for a “good” basis. B.- Distribution for a “bad” basis. The number of insertion orders available is *σ*! = 720. C.- Distribution of the 4,423,680 combinations of basis–insertion-order over CPU time.

The extreme point p = 0 of the system in Table 1 has |Z(p)|=20 and *σ* = 6. At this moderate level of degeneracy there is a set of 6144 bases, which represent a 16% of all possible ((2014)=38,760) choices and each basis may be accompanied by one of *σ*! = 720 different orders of the half-spaces in $B^{\prime }$. The 4,423,680 different choices of a basis B and an order for $B^{\prime }$ distribute over the CPU times as shown in Fig 5C. The spread of the distribution is $\bar{T}/\underline{T}=2.32$, where $\bar{T}$ and $\underline{T}$ are the CPU times for the worst and the best choices of a basis–insertion-order for the input pair $\left(B,\;B^{\prime }\right)$ in lines 5 and 6 of Algorithm 5. When choosing a basis B from Z(p) there are good and bad choices. A good basis has a narrow spread $\bar{T}/\underline{T}$ of insertion orders over the CPU times. Fig 5A is example of a good basis, with $\bar{T}/\underline{T}=1.27$. Fig 5B is the distribution for a bad basis having $\bar{T}/\underline{T}=2.10$. The difference between a good and a bad choice of the combination basis–insertion-order may represent in this example a factor greater than 2 in the CPU time.

**Fig 6:**
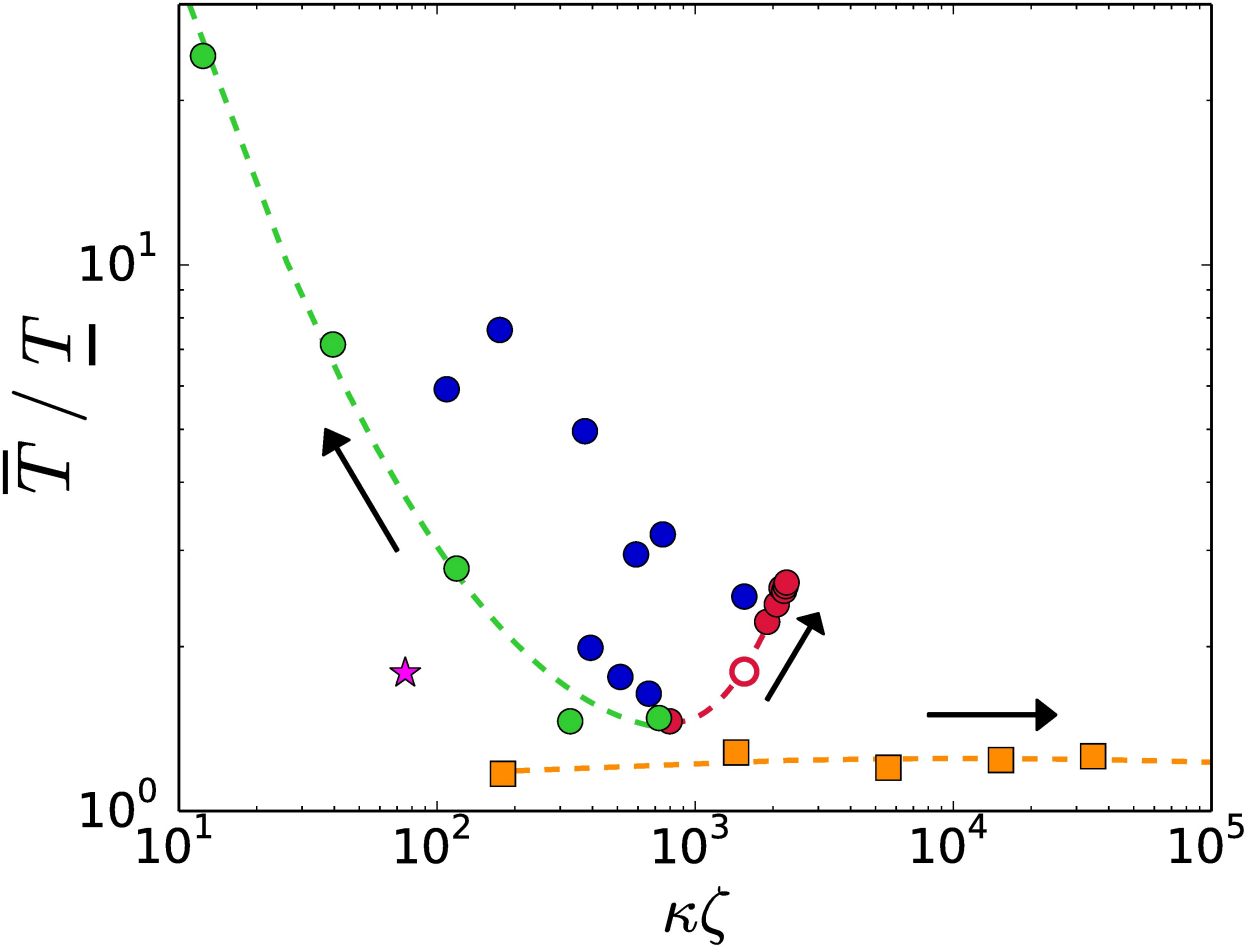
CPU time ratio of the worst ($\bar{T}$) and the best ($\underline{T}$) choices of basis–insertion-order. Sparsely-connected graphs show a higher sensitivity to the insertion order that those densely-connected.

A similar treatment for the three families of degenerate systems produces the distribution spreads $\bar{T}/\underline{T}$ shown in Fig 6, plotted as a function of the combined variable *κζ*. Degenerate systems that convert into densely connected graphs appear to have narrow spreads, $\bar{T}/\underline{T}\approx 1.2$ and $\bar{T}/\underline{T}<2.5$ for the k3 and k2 families, respectively. The choice of an insertion order is not an issue for them.

Degenerate systems that convert into bulky but sparsely-connected graphs show in Fig 6 a much higher sensitivity to the election of basis and insertion order. The spread $\bar{T}/\underline{T}$ of the dispersion of CPU times may be as wide as 24 for the cases used in our exploration and the spread keeps growing with *ζ*. Choosing a suitable combination of basis B and insertion order of $B^{\prime }$ is an issue for the optimization of the execution times of degenerate systems that convert into sparsely connected graphs.

**Fig 7:**
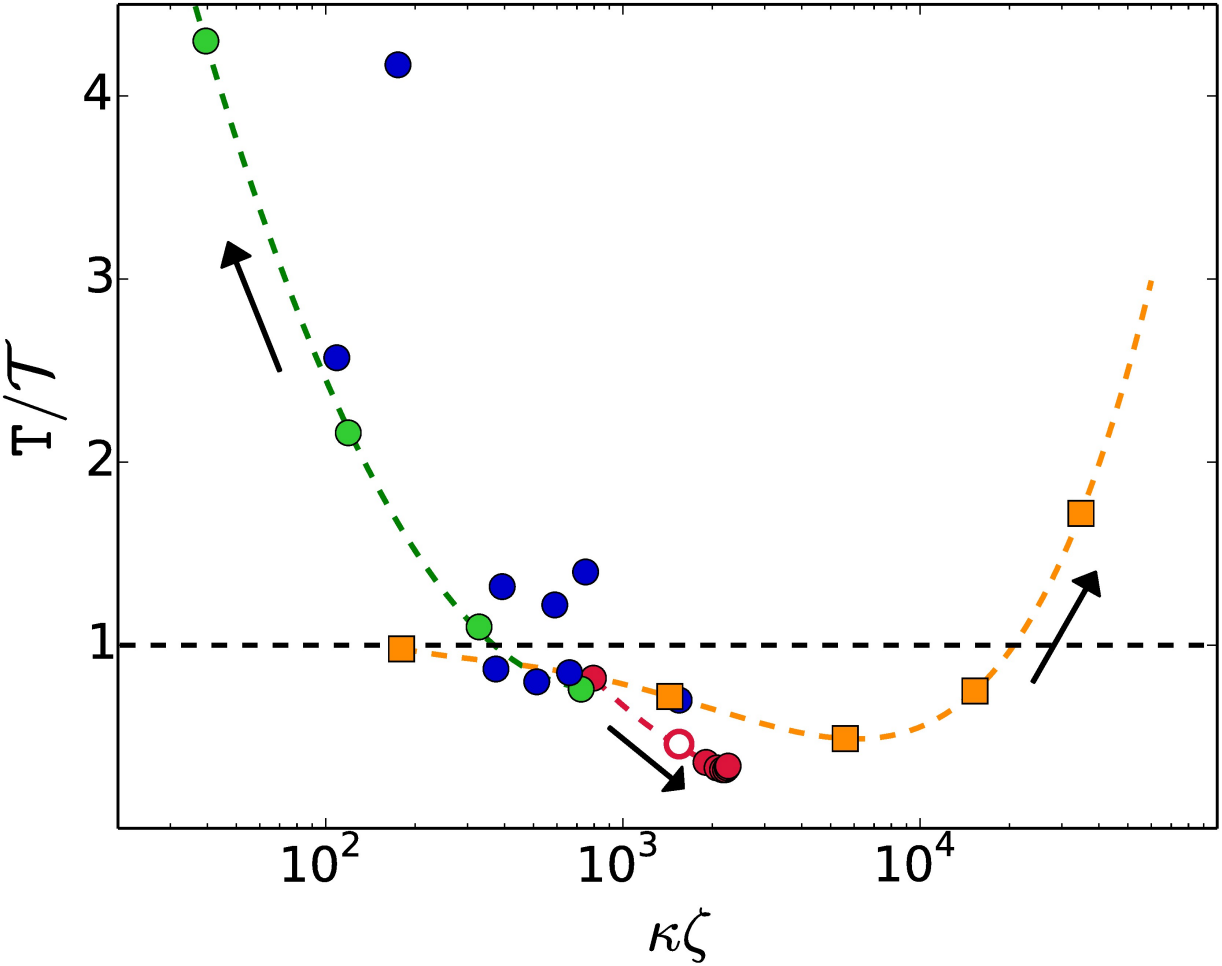
Ratio of CPU times for the combinatorial (T) and the algebraic (T) tests of 2-face cutting. The combinatorial test is the best option for systems that convert into densely-connected graphs while the algebraic test is appropriate for systems converting into sparsely-connected graphs.

The incremental slicing of cones may apply either a combinatorial or an algebraic test for 2-face cuttings. The results of a comparison of performance of the algebraic (T) and the combinatorial (T) tests are shown in Fig 7. The combinatorial test shows a better performance on systems that convert into densely connected graphs, the k2-family has the ratio T/T∼0.32. For systems that convert into sparsely connected graphs the algebraic test is the best option, specially for big input sizes. For the k3-family the combinatorial test is better for moderately bulky graphs, but when they get bigger the algebraic test turns out to be the better choice.

**Fig 8:**
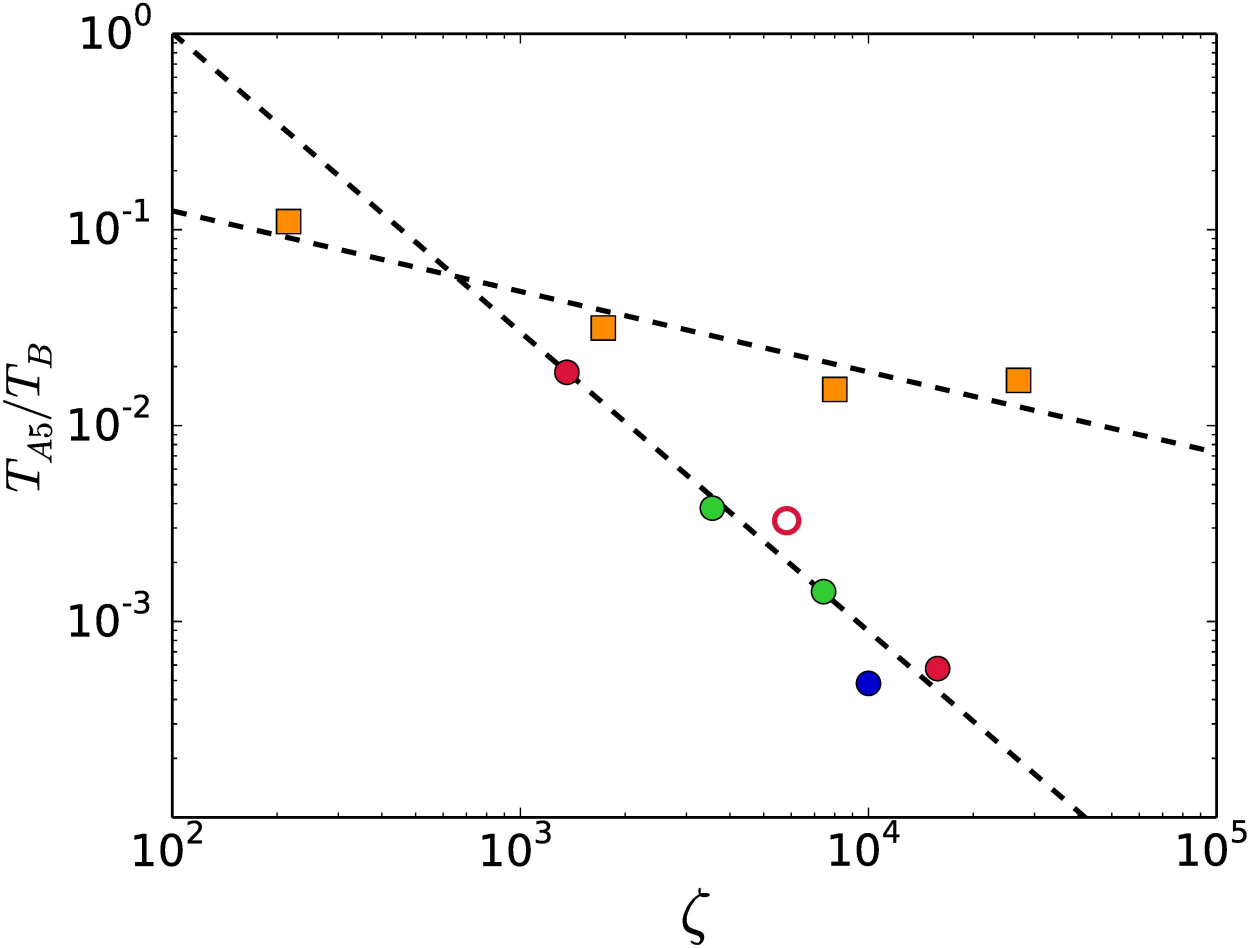
CPU time ratio *T*_A5_/*T*_*B*_. Algorithm 5 (*T*_A5_) is several orders of magnitud faster (polynomially) than our own implementation of Balinski’s algorithm (*T*_*B*_) [25].

Fig 8 compares the execution times of Algorithm 5 with the times employed by our own implementation of the pure-simplex based Balinski’s algoritm [25]. Only systems that were converted by Balinski’s algorithm in lesser than 50 hours are shown. Algorithm 5 performs several orders of magnitude better than Balinski’s. For instance the no-signaling system represented by the blue dot in Fig 8 was converted after 49 hours by Balinski’s algorithm, while Algorithm 5 took 86 seconds for the conversion.

## 6 Concluding remarks

The algorithm presented converts degenerate systems of linear inequalities into their skeleton graphs. Algorithm 5 applies an improved version of the incremental method for the enumeration of extreme rays to defeat degeneracy and a simple pivoting rule for a swift traversal of the set of extreme points.

The results obtained by Algorithm 5 in the computational practice of Section 5 characterize conversion problems in two classes, which are distinguished by the combined output-input measure of complexity *κζ*. Class A is constituted by systems of linear inequalities that convert degeneracy into densely connected graphs. Systems that convert degeneracy into bulky but sparsely connected graphs conform the second class B. The almost stationary value of *κζ* shown by the k2 family of no-signaling polytopes suggests that class B systems have values of *κζ* ≲ 10^3^.

The computational practice in Section 5 showed that Algorithm 5 performs better on systems of class A. For these systems, the incremental method is neither sensitive to the choice of the input basis nor to the insertion order of the cutting half-spaces and there is a clear evidence that favors the combinatorial over the algebraic 2-face test.

On the contrary, for class B systems the performance of the incremental method is highly sensitive to the large number of combinatorial options introduced by degeneracy. The selection of an “optimal” input basis and an “optimal” insertion order remains a problem for the systems in class B. No solution to this problem is found in the literature, only recommendations to achieve some technical easiness, such as keeping a fixed insertion order and the remark that any dynamical reordering based on explorations consumes a longer CPU time [19, 29, 31]. The recommendation for class B derived from the computational practice of Section 5 is to explore a few extreme points in advance as to find a good order of the constraint vectors for the sample of points and then adopt the finding for the whole procedure.

Concerning the 2-face test, the algebraic one is undoubtedly the best option for class B systems. In practice, it is advisable to run a competition of the combinatorial and algebraic tests on a small sample of extreme points and use the winning test to proceed with the full conversion.

In the extensive practice of Section 5, Algorithm 5 showed a superior performance respect the simplex-based pivoting algorithm by Balinski [25]. Our own implementation of Balinski’s algorithm got stalled (did not finish) when trying to convert most of the systems studied in Section 5.

In this paper we have used the combined output-input variable *κζ* as the measure of complexity for the output graphs. However, there exist other measures for characterizing such complexity, as the graph entropy [13, 14] and the graph similarity [15]. Then, a future work that involves such measures to characterize the output graphs produced by degenerate systems of linear restrictions would be interesting.

The algorithm introduced in this paper aims to be a useful tool in applied problems that require a conversion mechanism for an appropriate interpretation. We are aware that a theoretical analysis of its computational complexity is required. However, for an analysis of computational complexity to be of some significance, a precise and definite characterization of the input descriptions is required. The problem is that, unlike regular input systems, degenerate graphs cannot be described simply by their dimension and number of half-spaces, as shown in Section 5. We did not find in the literature a standard characterization for the input descriptions as to typify degenerate systems. The no-signaling and Birkhoff’s half-space descriptions are candidates to consider, but first one of them should be characterized as the specific standard for degeneracy. On the other hand, the effect of implementations that optimize the performance of the algorithm (such as the 2-face recording and the rejection test) must be differentiated and evaluated too. The above considerations tells us that, even necessary, a significant analysis of computational complexity is a very ambitious endeavor that lays beyond the scope of this article.

## References

[1] Bell JS, On the Einstein Podolsky Rosen paradox. *Physics* 1, 195–200 (1964).

[2] Popescu S, Rohrlich D. Nonlocality as an axiom. *Foundations of Physics* 24, 379–385 (1994).

[3] Barrett J, Pironio S. Popescu-Rohrlich correlations as a unit of nonlocality. *Phys. Rev. Lett*. 95, 140401 (2005). 10.1103/PhysRevLett.95.140401 16241631

[4] Barrett J. et al, Nonlocal correlations as an information-theoretic resource. *Phys. Rev. A* 71, 022101 (2005). 10.1103/PhysRevA.71.022101

[5] Cerf NJ, Gisin N, Massar S, Popescu S. Simulating maximal quantum entanglement without communication. *Phys. Rev. Lett*. 94, 220403 (2005). 10.1103/PhysRevLett.94.220403 16090370

[6] Pironio S, Bancal JD, Scarani V. Extremal correlations of the tripartite no-signaling polytope. *J. Phys. A: Math. Theor*. 44, 065303 (2011). 10.1088/1751-8113/44/6/065303

[7] Méndez JM, Urías J. On the no-signaling approach to quantum nonlocality. *J. Math. Phys*. 56, 032101 (2015).

[8] Stelling J, Klamt S, Bettenbrock K, Schuster S, Gilles E. D. Metabolic network structure determines key aspects of functionality and regulation. *Nature* 420, 190 –193 (2002). 10.1038/nature01166 12432396

[9] Wang Z, Gao H, Cao J, Liu X. On delayed genetic regulatory networks with polytopic uncertainties: robust stability analysis. *IEEE Transactions on NanoBioscience*, Vol. 7, No. 2, 6 2008 10.1109/TNB.2008.2000746 18556263

[10] Amaris AJR, Cox MP. A flexible theoretical representation for the temporal dynamics of structured populations as paths on polytope complexes. *J. Math. Biol*. 71, 735–766 (2015). 10.1007/s00285-014-0841-4 25307774 PMC4532729

[11] Hart Y. et al, Inferring biological tasks using Pareto analysis of high-dimensional data. *Nature Methods*, Vol. 12, No. 3, 3 2015, pp. 233–235. 10.1038/nmeth.3254 25622107

[12] Gagneur J, Klamt S. Computation of elementary modes: A unifying framework and the new binary approach. *BMC Bioinformatics* 5, 175 (2004). 10.1186/1471-2105-5-175 15527509 PMC544875

[13] Cao S, Dehmer M, Shi Y. Extremality of degree-based graph entropies. *Inform. Sci*. 278, 22–33 (2014). 10.1016/j.ins.2014.03.133

[14] Chen Z, Dehmer M, Emmert-Streib F, Shi Y. Entropy of weighted graphs with Randic weights. *Entropy* 17(6), 3710–3723 (2015).

[15] Emmert-Streib F, Dehmer M, Shi Y. Fifty years of graph matching, network alignment and comparison. *Inform. Sci*. 346–347, 180–197 (2016). 10.1016/j.ins.2016.01.074

[16] Avis D, Fukuda K. A pivoting algorithm for convex hulls and vertex enumeration of arrangements and polyhedra. *Discrete Comput Geom* 8, 295–313 (1992). 10.1007/BF02293050

[17] Bremner D, Fukuda K, Marzetta A. Primal-dual methods for vertex and facet enumeration. *Discrete Comput Geom* 20, 333–357 (1998). 10.1007/PL00009389

[18] Motzkin TS, Raiffa H, Thompson GL, Thrall RM. The double description method Appeared in *Contributions To The Theory Of Games* Vol. 2, (ed. Kuhn H. W. & Tucker A. W.) Princeton University Press (1971). 10.1515/9781400881970-004

[19] Fukuda K, Prodon A. Double description method revisited. *Combinatorics Comp. Sci*. 1120, 91–111 (1996). 10.1007/3-540-61576-8_77

[20] Dyer ME, Proll L. G. An algorithm for determining all extreme points of a convex polytope. *Math. Program*. 12, 81–96 (1977). 10.1007/BF01593771

[21] Gal T. On the structure of the set bases of a degenerate point. *J. Optim. Theory Appl*. 45, 577–589 (1985). 10.1007/BF00939135

[22] Gal T, Geue F. A new pivoting rule for solving various degeneracy problems. *Ops. Res. Lett*. 11, 23–32 (1992). 10.1016/0167-6377(92)90058-B

[23] Yamada T, Yoruzuya J, Kataoka S. Enumerating extreme points of a highly degenerate polytope. *Computers Ops. Res*. 21, No. 4, pp. 397–410 (1994). 10.1016/0305-0548(94)90027-2

[24] Dantzig GB, Orden A, Wolfe P. The generalized simplex method for minimizing a linear form under linear inequality restraints. *Pacific J. Math*. 5, 183–195 (1955). 10.2140/pjm.1955.5.183

[25] Balinski ML. An algorithm for finding all vertices of convex polyhedral sets. *J. SIAM* 9, 72–88 (1961).

[26] Avis D, Bremner D, Seidel R. How good are convex hull algorithms?. *Comput. Geom*. 7, 265–301 (1997). 10.1016/S0925-7721(96)00023-5

[27] Brualdi RA. Combinatorial matrix theory Appeared in *Handbook Of Linear Algebra*, Second Edition (Discrete Mathematics and Its Applications), (ed. Hogben L.) Chapman & Hall/CRC (2014). 10.1201/9781420010572.pt2

[28] Pak I. Four questions on Birkhoff polytope. *Annals of Combinatorics* 4, 83–90 (2000). 10.1007/PL00001277

[29] Zolotykh NY. New modification of the double description method for constructing the skeleton of a polyhedral cone. *Comput. Math. and Math. Phys*. 52, 146–156 (2012). 10.1134/S0965542512010162

[30] McMullen P. The maximum numbers of faces of a convex polytope. *Mathematika* 17, 179–184 (1970).

[31] Martí R, Reinelt G. The Linear Ordering Problem: Exact and Heuristic Methods in Combinatorial Optimization Applied Mathematical Sciences, Vol. 175, Springer (2011).

